# Two decades of FDG-PET/CT in seminoma: exploring its role in diagnosis, surveillance and follow-up

**DOI:** 10.1186/s40644-022-00496-w

**Published:** 2022-10-08

**Authors:** Ciara Conduit, Thuan Tzen Koh, Michael S Hofman, Guy C Toner, Jeremy Goad, Nathan Lawrentschuk, Keen-Hun Tai, Jeremy H Lewin, Ben Tran

**Affiliations:** 1 Department of Medical Oncology, Peter MacCallum Cancer Centre, 305 Grattan St, 3031 Melbourne, VIC Australia; 2 Sir Peter MacCallum Department of Oncology, The University of Melbourne, Parkville, VIC Australia; 3 Walter and Eliza Hall Institute of Medical Research, Melbourne, VIC Australia; 4 Molecular Imaging and Therapeutic Nuclear Medicine, Cancer Imaging, Peter MacCallum Cancer Centre, Melbourne, VIC Australia; 5 Department of Radiology and Nuclear Medicine, Flinders Medical Centre, Adelaide, SA Australia; 6 Department of Cancer Surgery, Peter MacCallum Cancer Centre, Melbourne, VIC Australia; 7 Department of Surgery, University of Melbourne, Royal Melbourne Hospital, Melbourne, Australia VIC; 8 Department of Radiation Oncology, Peter MacCallum Cancer Centre, Melbourne, VIC Australia; 9 ONTrac at Peter Mac, Victorian Adolescence and Young Adult Cancer Service, Melbourne, Australia VIC

**Keywords:** Testicular neoplasm, Seminomas, cancer survivors, PET-CT scan, Biomarkers

## Abstract

**Background:**

Survivors of testicular cancer may experience long-term morbidity following treatment. There is an unmet need to investigate techniques that can differentiate individuals who need additional therapy from those who do not. 2-^18^fluoro-deoxy-D-glucose (FDG) positron emission tomography (PET) with computerised tomography (CT) may be helpful in select settings and may be used outside of current evidence-based recommendations in real-world practice.

**Methods:**

A institutional FDG-PET/CT database of scans performed between 2000 and 2020 for adults with testicular seminoma was interrogated. Endpoints of interest included the positive (PPV) and negative (NPV) predictive value of FDG-PET/CT for identifying active seminoma (defined by progressive radiology, response to treatment or biopsy); or no active seminoma within 24-months for patients with stage 1 and advanced seminoma. An exploratory analysis examining predictive role of SUV_max_ was also performed.

**Results:**

249 patients met eligibility criteria for the analysis, including 184 patients with stage 1 and 77 patients with advanced testicular seminoma. Of 193 FDG-PET/CT performed in stage 1 seminoma with available follow-up data, 79 were performed during active surveillance. 18 (23%) of these were positive, all of which had confirmed recurrent seminoma (PPV 100%). Of 45 negative FDG-PET/CT during active surveillance, 4 recurrences developed corresponding to a NPV 91%. When clinical suspicion precipitated FDG-PET/CT (n = 36): PPV 100%, NPV 86%. Of 145 FDG-PET/CT in advanced seminoma with available follow-up data, 25 (17%) were performed at baseline (within 2 months of diagnosis), 70 (48%) post-treatment for evaluation of treatment response and 50 (34%) during follow-up following prior curative treatment. 10 (14%) post-treatment FDG-PET/CT were positive corresponding to a PPV 60%. Of 46 negative FDG-PET/CT, 5 recurrences occurred (NPV 89%). During follow-up after prior curative treatment, 24 (50%) FDG-PET/CT were positive corresponding to a PPV 83%; of 20 negative FDG-PET/CT, 1 recurrence occurred, NPV 95%. When clinical suspicion indicated FDG-PET/CT (n = 36): PPV 100%, NPV 94%.

**Conclusion:**

FDG-PET/CT offers high PPV for identifying seminoma and accurately predicts non-recurrence across a clinically relevant 24-months. Notably, FDG-PET/CT may prevent unnecessary treatment in 45% of patients undergoing investigation for clinical suspicion of recurrence during follow-up of advanced seminoma. The use of FDG-PET/CT in selected patients now, may help prevent unnecessary treatment of people with testicular seminoma.

**Supplementary Information:**

The online version contains supplementary material available at 10.1186/s40644-022-00496-w.

## Introduction

Testicular cancer is curable in most individuals, regardless of histologic subtype, disease stage or primary site, owing to significant advances in treatment [1]. However, there is growing evidence that survivors experience significant long-term morbidity [2–4] and impaired health-related quality of life related to treatment [5, 6]. There is an unmet need to investigate novel techniques that can differentiate those who need additional therapy to secure cure from those who do not.

2-^18^fluoro-deoxy-D-glucose (FDG) positron emission tomography (PET) with computerised tomography (CT) is indicated in the evaluation of residual masses ≥ 3 cm following chemotherapy for testicular seminoma [7, 8]. Unlike dedicated CT imaging, which cannot differentiate between treatment-related fibrosis/necrosis and residual seminoma in this setting, FDG-PET/CT detects viable residual tumour with high positive (PPV) and negative predictive value (NPV) when performed six weeks after chemotherapy as demonstrated in a prospective dataset [7]. With this approach, FDG-PET/CT prevents unnecessary additional treatment in up to 70% of individuals and is endorsed in international guidelines [1, 9, 10]. Outside of this context however, FDG-PET/CT is not routinely recommended for evaluation of seminoma, despite early evidence supporting its use across multiple areas [11–19], nor in non-seminoma where lower diagnostic accuracy has been reported [12, 20].

Notwithstanding lack of endorsement and limited prospective data, FDG-PET/CT is anecdotally performed as part of active surveillance, evaluation of non-specific imaging findings and treatment response in the “real-world” management of testicular seminoma. We explored its clinical utility in the management of testicular seminoma through a retrospective review of FDG-PET/CT conducted in a real-world setting.

## Patients and methods

An institutional database was interrogated to identify adults with testicular seminoma who underwent FDG-PET/CT between 2000 and 2020 extracting demographic, clinicopathological, FDG-PET/CT findings and outcome data. Ethical approval was granted by the Melbourne Health Human Research Ethics Committee. Patients with non-seminoma/mixed germ cell tumours, bilateral testicular malignancy and extragonadal (or unknown) primary sites were excluded.

When FDG-PET/CT was reported as consistent with seminoma (positive) and maximum standardised uptake value (SUV_max_) was unavailable in the clinical report, the study was reviewed again by a dual-trained nuclear medicine physician and radiologist (TK) to confirm presence of a target lesion. Target lesions were selected using the Response Evaluation Criteria in Solid Tumours (RECIST), v1.1 [21]. Where possible, measurable lesions/lymph nodes were preferred, however in the absence of a measurable target lesion, a non-measurable lesion/lymph node was selected using the same criteria. Once a target lesion was identified, the SUV_max_ value was measured.

In the absence of validated objective criteria for evaluating FDG-PET treatment/other response in seminoma, the PPV of FDG-PET/CT for correctly identifying seminoma (true positive) was pragmatically defined as progressive radiological change of target lesion(s) on serial imaging, evidence of response to definitive treatment, or histological confirmation. The NPV for correctly identifying no evidence of seminoma (or non-recurrence, true negative) at 24-months following FDG-PET/CT was calculated. 24-months was considered a clinically relevant period to demonstrate clinical utility. Exploratory analyses of SUV_max_ cut-offs were undertaken.

**Fig. 1:**
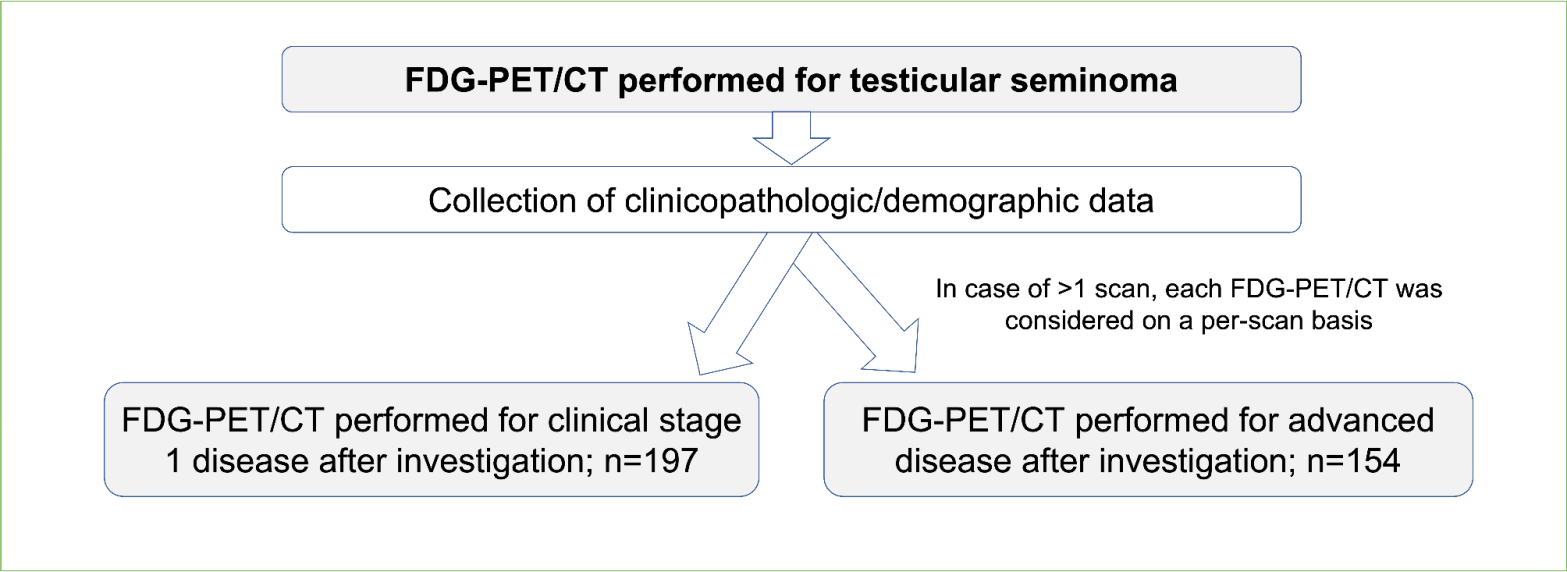
Overall Study Schema. (PPV = positive predictive value, NPV = negative predictive value, FDG-PET/CT = 2-^18^fluoro-deoxy-D-glucose positron emission tomography with computerised tomography)

To preserve clinical relevance, FDG-PET/CT were analysed in cohorts defined by clinical stage at the time FDG-PET/CT was performed (per the AJCC, 8th edition) [22]. The two cohorts were: ‘stage 1’ and ‘advanced’ testicular seminoma. Patients were considered to have stage 1 seminoma where investigation results, including FDG-PET/CT (if performed at baseline), suggested seminoma was confined to the testis. Patients were considered to have advanced seminoma if investigation results were consistent with clinical stage 2 or 3 disease (see Figure 1). The indication for FDG-PET/CT was recorded and defined as ‘routine’, when performed as part of the clinician’s regular practice; ‘clinical suspicion’ where existing radiological investigations, serum tumour biomarkers or symptoms indicated the scan; or ‘post-treatment’, which was indicated for evaluation of treatment response, i.e., response to definitive chemotherapy. A ‘baseline’ scan was defined as a FDG-PET/CT performed within two months of diagnosis; all other scans were considered ‘follow-up.’ If > 1 FDG-PET/CT was performed per individual, each FDG-PET/CT was treated on a per-scan basis, which allowed for follow-up and calculation of PPV/NPV for each scan at 24-months. Patients were censored from predictive value analyses if they had insufficient follow-up data.

## Results

Between 2000 and 2020, 249 patients met eligibility criteria for the analysis, including 184 patients with stage 1 and 77 patients with advanced testicular seminoma (inclusive of 12 individuals who underwent FDG-PET/CT for stage 1 and advanced seminoma) (see Figure 1). Baseline clinical and demographic data is displayed in Table 1. Almost two thirds (63%) of patients in the stage 1 cohort received adjuvant radiation therapy (n = 82, 45%) or chemotherapy (n = 33, 18%) following orchidectomy. Of the patients in the advanced stage cohort who had relapsed stage 1 disease, prior adjuvant chemotherapy or radiation therapy was received by 38%.

**Table 1:** Characteristics of Patients at Baseline

		Stage 1 (n = 181)	Advanced (n = 77)
**Median age, years (range)**		36 (14–64)	38 (14–61)
**Initial clinical staging, n (%)**	Localised	184 (100)	15 (19)
	Advanced	-	62 (81)
**Surgical management, n (%)**	Orchidectomy	184 (100)	71 (92)
	Retroperitoneal lymph node dissection	-	2 (3)
	Other surgery (metastectomy)	-	2 (3)
**Adjuvant therapy for stage 1 seminoma, n (%)**	Chemotherapy	33 (18)	3 (20)*
	Radiation therapy	82 (45)	3 (20)*
	None	63 (34)	9 (60)*
	Unknown	6 (3)	0 (0)*
**Treatment(s) for advanced/recurrent testicular seminoma, n (%)**	Chemotherapy	-	43 (56)
	Radiation therapy	-	40 (52)
	Surgery	-	4 (5)
**Number of FDG-PET/CT per patient, n (range)**		1 (1–3)	2 (1–7)
**Median follow-up from initial diagnosis with seminoma, years (range)**		6.4 (0–17)	9.3 (0–25)

*Of 15 individuals originally diagnosed with stage 1 seminoma and eligible for adjuvant treatment

Follow-up continued for a median of 6.4 years (range 0–17) from diagnosis for patients in the stage 1 cohort, and 27 (15%) recurrences occurred. The median time from diagnosis to FDG-PET/CT was 1.7 months (range 0-121), the median number of FDG-PET/CT performed at diagnosis or during active surveillance was 1 (range 1–3).

In contrast, patients in the advanced cohort were followed for a median of 7.7 years (range 0–25) from original diagnosis (with initial stage 1 or de novo advanced disease), and 28 (36%) episodes of relapsed/refractory disease were observed (including 12 individuals or 16%, initially evaluated in the stage 1 cohort). The median time from diagnosis to first FDG-PET/CT was 9.1 months (range 0-166) and the median number of FDG-PET/CT performed for evaluation of advanced seminoma was 2 (range 1–7).

### Stage 1 cohort

**Fig. 2:**
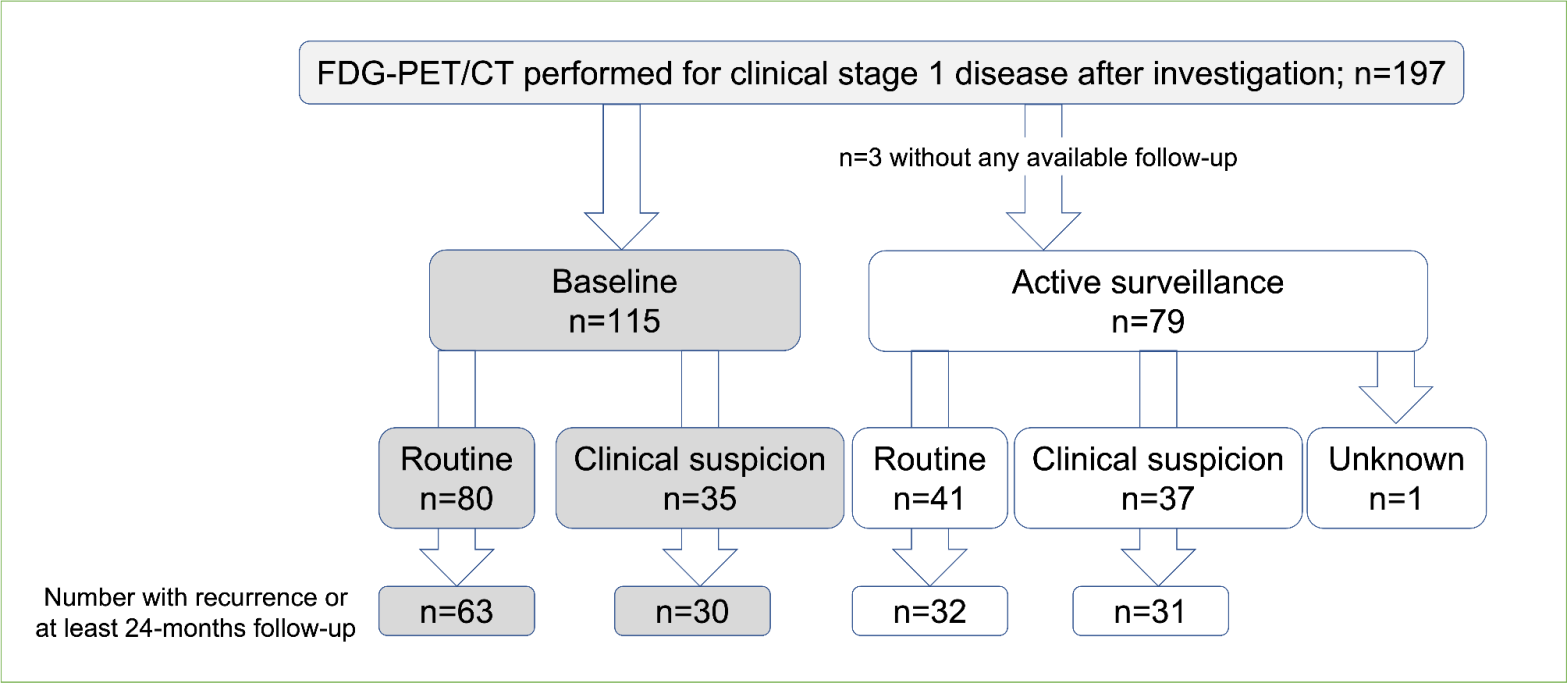
Evaluable FDG-PET/CT performed for clinical stage 1 seminoma. (PPV = positive predictive value, NPV = negative predictive value, FDG-PET/CT = 2-^18^fluoro-deoxy-D-glucose positron emission tomography with computerised tomography)

In patients with stage 1 seminoma, 197 FDG-PET/CT were performed, however three FDG-PET/CT had no available follow-up data and were excluded from further analyses. Of the remaining 194 FDG-PET/CT, 115 (60%) were performed at baseline and the remaining 79 (41%) were performed during active surveillance. The most common indication for FDG-PET/CT was as a ‘routine’ component of clinical practice during diagnostic workup (n = 80, 41%) or active surveillance (n = 41, 21%) (see Figure 2).

Of the 115 baseline FDG-PET/CT, all were negative, and 93 had ≥ 24 months of follow-up during which time, four recurrences occurred (in four patients). The NPV for FDG-PET/CT predicting non-recurrence at 24 months was 95.7% (see Table 2 and Supplementary Table 1). Of 78 FDG-PET/CT ordered during active surveillance with available follow-up, 18 (23%) were reported as positive with recurrence confirmed in all cases following apparent radiologic response after definitive treatment (PPV 100%). Of the 45 active surveillance FDG-PET/CT reported as negative with ≥ 24 months of follow-up, four episodes of recurrence occurred in four patients (NPV 91.1%). The median time-to-recurrence following negative FDG-PET/CT was 1.6 years (range 1–4).

**Table 2:** Positive and negative predictive values for FDG-PET/CT by indication at 24-months

Cohort	Setting	Indication	Positive predictive value (%)	Negative predictive value at 24-months (%)
Stage 1	Baseline	Total: Routine or clinical suspicion^*#*^	NA	95.7
	Active surveillance	Total: Routine, clinical suspicion or unknown	100	91.1
		Routine	100	93.6
		Clinical suspicion^*#*^	100	85.7
Advanced	Baseline	Total: Routine or clinical suspicion^*#*^	100	0
	Follow-up	Total: Routine, clinical suspicion^*#*^or post-treatment	77.1	90.9
		Routine	42.9	100
		Clinical suspicion^*#*^	100	93.8
		Post-treatment*	60	89.1

^#^including radiological abnormality on existing imaging, elevation of serum tumour markers or symptoms.

*Inclusive of patients with and without residual mass post-chemotherapy.

When indicated by clinical suspicion during active surveillance (n = 37), inclusive of abnormal radiology (n = 32), elevated serum tumour markers (n = 4) and symptoms (n = 1), 17 FDG-PET/CT were reported as “positive” for recurrent seminoma. All were true positives (PPV 100%) evidenced by radiologic response to definitive treatment. Of those with ≥ 24 months follow-up in this cohort, the NPV was 85.7% (two recurrences amongst 14 patients). In real terms, the use of FDG-PET/CT in patients with stage 1 seminoma in this setting correctly identified 12/37 patients who did not have recurrence (12/14 FDG-PET/CT reported as negative), and correspondingly, may prevent unnecessary treatment in ~ 1/3 individuals undergoing FDG-PET/CT for this indication.

### Advanced stage cohort

**Fig. 3:**
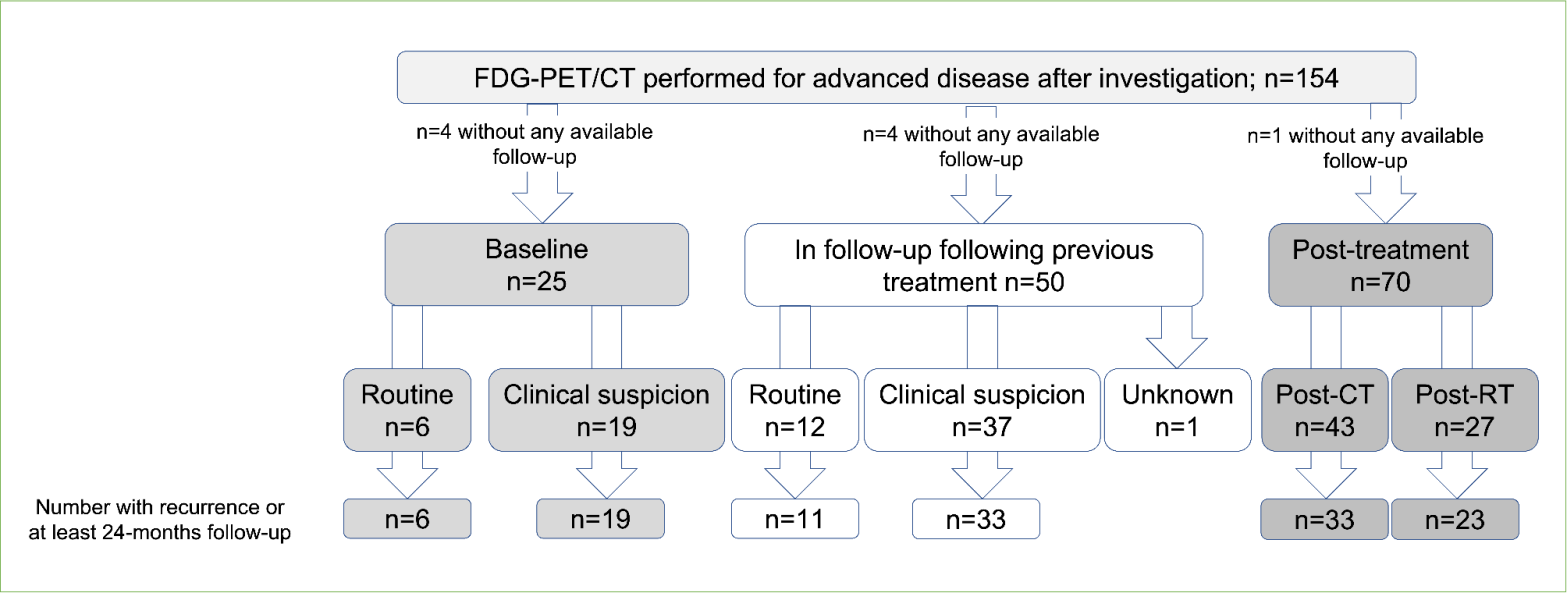
Evaluable FDG-PET/CT performed for advanced seminoma. (PPV = positive predictive value, NPV = negative predictive value, FDG-PET/CT = 2-^18^fluoro-deoxy-D-glucose positron emission tomography with computerised tomography)

In patients with advanced seminoma, 154 FDG-PET/CT were performed, however nine FDG-PET/CT had no available follow-up data and were excluded from all further analyses. Of the remaining 145 FDG-PET/CT, 25 (17%) occurred at baseline. 70 (48%) were performed following chemotherapy or radiation therapy (to evaluate response), 50 (34%) were performed in follow-up after prior curative treatment of advanced seminoma. The most common indication for FDG-PET/CT was investigation of abnormal radiology at baseline or during follow-up (n = 49, 32%), or immediately following chemotherapy to assess response (n = 44, 29%) (see Figure 3).

Overall, 24/25 baseline scans were reported as positive (96%), and active disease was confirmed in all cases (PPV 100%). Of these, two (8%) were confirmed on histology, with the remainder confirmed on follow-up radiology after treatment. One baseline FDG-PET/CT performed for investigation of abnormal radiology was reported as negative; however, this individual received definitive radiation therapy and sustained a complete response, and the finding was considered a false negative (see Table 2).

When FDG-PET/CT was ordered following chemotherapy or radiation therapy to evaluate response, 10 (14%) were reported as positive and residual active disease was confirmed in six cases corresponding to a PPV of 60.0%. Of the 46 FDG-PET/CT reported as negative with ≥ 24 months of follow-up, five episodes of recurrence occurred in five patients (NPV 89.1%). The presence and size of any residual mass triggering FDG-PET/CT was not available. The median time-to-recurrence following the negative FDG-PET/CT was 11 months (range 6–20). As such, of the 71 FDG-PET/CT performed following definitive treatment, 41 (58%) correctly identified no residual seminoma and avoided unnecessary treatment.

In an evaluation of 50 FDG-PET/CT performed during follow-up of prior curative treatment of advanced testicular seminoma (inclusive of FDG-PET/CT ordered routinely and for clinical suspicion of recurrent disease), one patient had an equivocal FDG-PET/CT result leaving 49 FDG-PET/CT in this analysis. Of these, 24 (49%) were reported as positive and recurrence was confirmed in 20 cases following additional definitive treatment (PPV 83%). Of the 20 FDG-PET/CT reported as negative with ≥ 24 months of follow-up, one episode of disease recurrence occurred, corresponding to a NPV of 95%. Notably, this patient experienced intracranial relapse detected on magnetic resonance imaging three weeks following FDG-PET/CT.

When indicated by clinical suspicion alone (n = 36), inclusive of abnormal radiology (n = 25), elevated serum tumour markers (n = 5) and symptoms (n = 7) during follow-up, PPV was 100% while NPV was 93.8% in those with ≥ 24 months follow up (1 recurrence amongst 16 patients). Practically, the use of FDG-PET/CT in patients with advanced seminoma who had clinical suspicion of recurrence following definitive treatment, correctly identified 15 patients who did not experience recurrence and correspondingly, prevented unnecessary treatment being given to 45% of patients.

### Exploration of SUV_max_

Of the 90 FDG-PET/CT reported as consistent with active seminoma, SUV_max_ was available for 19 (86%) in the stage 1 cohort, and 34 (50%) in the advanced disease cohort. There was no difference in mean SUV_max_ between stage 1 and advanced cohorts (9.33 versus 8.52, *p* = 0.65). When analysed together by recurrence status (true positive versus false positive), higher SUV_max_ was more frequently seen in patients with seminoma than no active malignancy (9.56 *versus* 5.10, *p* = 0.14), however this did not meet statistical significance. Utilising aggregated SUV_max_ data, the PPV of SUV_max_ when greater than the 1st interquartile range (IQR) (SUV_max_>4.7, n = 34), median (SUV_max_>6, n = 25) and 3rd IQR (SUV_max_>11.9, n = 12) was 91%, 96% and 100%, demonstrating higher predictive value associated with higher SUV_max_ cut-offs.

## Discussion

There is an unmet need to develop new techniques to accurately identify patients with seminoma who do not require additional treatment. In our study, FDG-PET/CT served as a useful adjunct to existing tools, allowing clinicians to accurately diagnose recurrence with high PPV in most clinical scenarios, and importantly, predict non-recurrence across a clinically relevant period of 24-months. FDG-PET/CT is available in highly resourced settings like ours and may be used to guide the management of patients with seminoma in *select* circumstances whilst novel tools are awaited [23–26].

The introduction of FDG-PET/CT into routine care has led to a paradigm shift in oncology [27]. This is especially true of malignancies with high metabolic rates, where the radiotracer, FDG, becomes trapped in malignant cells secondary to upregulation of GLUT transporters [28]. Despite widespread acceptance of FDG-PET/CT in other tumour types, the only validated indication for the investigation within testicular cancer is in evaluation of residual post-chemotherapy masses following definitive treatment of advanced seminoma [7]. However, use of the investigation in this setting appears to have limitations in the real-world where lower sensitivity, specificity and PPV have been reported [29]. There is also no established role in other clinical scenarios, however evidence of its potential utility in safely de-escalating chemotherapy in advanced seminoma is emerging [17]. Additionally, access to FDG-PET/CT varies worldwide due to availability and issues of reimbursement, limiting its applicability outside highly-resourced settings [30]. In non-seminoma, comparatively low FDG avidity, particularly when teratoma is present, has limited its clinical utility [31, 32] and FDG-PET/CT is therefore not recommended in patients with non-seminoma. Outside of FDG-PET/CT, other emerging tools such as micro-ribonucleic acids (miRNA) may also serve as useful adjuncts in both seminoma and non-seminoma in the future [24–26, 33]. Ongoing trials to elucidate the role for miR-371 in the clinic are likely to cement miR-371 into the diagnostic and management paradigm [34].

Data from our study suggest that FDG-PET/CT may have similar discriminatory accuracy to miR-371 demonstrated in early studies [26]. In our stage 1 cohort, the NPV was 86% when FDG-PET/CT was ordered for suspicion of recurrence during active surveillance. Notwithstanding that a large proportion of the cohort received adjuvant therapy (largely reflecting enrolment prior to 2010) and were at lower risk of recurrence than patients entering active surveillance after orchidectomy, a high NPV offers some reassurance for patients and clinicians in this setting. Of course, the risks associated with over-treatment and over-investigation also needs to be considered in this cohort of individuals with otherwise excellent survival and despite reassuring results, routine application of FDG-PET/CT as a component of active surveillance cannot be recommended in patients with stage 1 seminoma.

In individuals with advanced disease, FDG-PET/CT had a lower PPV (range 43–100%), however importantly the clinically relevant NPV for predicting non-recurrence at 24-months generally remained high (range 89–100%). When miR-371 was evaluated in a similar context, inclusive of both patients with seminoma and non-seminoma, it offered an 89% PPV and comparatively low, 67% NPV for predicting non-recurrence across a median 15-month follow-up [26]. While our data suggests that FDG-PET/CT may not be quite as accurate as miR-371 at identifying recurrence or predicting non-recurrence, FDG-PET/CT has the advantage of being available. In our institution’s experience and with the benefit of prolonged follow-up, FDG-PET/CT can prevent unnecessary treatment in 38% of patients in follow-up after prior definitive treatment of advanced seminoma. Further investigation into the value of SUV_max_ cut-offs may be warranted.

In our population, 58% of patients undergoing FDG-PET/CT following definitive treatment could also safely avoid further treatment. Notably however, the PPV was low in this population, which may be explained by short interval follow-up after treatment completion in some individuals (i.e., prior to six weeks) [29]; a datapoint which was unavailable due to administration of definitive treatment at external centres in some patients and referral in for FDG-PET/CT only. However, our study did not seek to validate prior findings of the role for FDG-PET/CT in this context, which is already well-established [1, 9, 10].

This retrospective, single-centre analysis describes the largest known series of FDG-PET/CT performed for testicular seminoma. Our cohort of high-risk advanced seminoma patients treated at a specialist cancer centre may account for the high rates of relapse/refractory disease. Owing to disparities in access to FDG-PET/CT worldwide and variability in practice, this data has limited generalisability outside highly resourced settings and recruitment across a twenty-year period introduces heterogeneity and biases. Whilst PET technology has evolved over the last 20 years, most studies were acquired at a single centre with robust quality control per the European Association of Nuclear Medicine FDG-PET/CT guideline [35], including SUV standardisation across cameras. It was not within the scope of this research to analyse inter-reader agreement and instead we set out to determine the patterns of use of FDG-PET/CT in a real-world environment. Other limitations include missing data regarding disease volume (including post-chemotherapy residual masses), lack of central review of FDG-PET/CT results and how results may have impacted clinical decision-making.

## Conclusion

FDG-PET/CT is a useful adjunct to existing tools and allows clinicians to accurately detect seminoma and importantly, predict non-recurrence across a clinically relevant period. The use of FDG-PET/CT in selected patients, may help prevent unnecessary treatment of men with testicular seminoma, particularly in those where recurrence is suspected. In turn, we can shift the focus away from treatment and to the quality of survival for this growing population.

## Electronic supplementary material

Below is the link to the electronic supplementary material.

Supplementary table 1

## Data Availability

The datasets used and/or analysed during the current study are available from the corresponding author on reasonable request.

## Abbreviations

PPV: positive predictive value.
NPV: negative predictive value.
FDG-PET/CT: 2-^18^fluoro-deoxy-D-glucose positron emission tomography with computerised tomography.
FDG: 2-^18^fluoro-deoxy-D-glucose.
SUV_max_: maximised standardised uptake value.
AJCC: American Joint Classification on Cancer.
miRNA: microribonucleic acids.
