# Supplementary material for: Two decades of FDG-PET/CT in seminoma: exploring its role in diagnosis, surveillance and follow-up
Source: Cancer Imaging. 2022 Oct 8;22:58. doi: 10.1186/s40644-022-00496-w (PMC9548159; doi:10.1186/s40644-022-00496-w)
Supplement: Supplementary file 1 — Supplementary table 1 [file 40644_2022_496_MOESM1_ESM.docx]

**Supplementary Table I:** Positive and negative predictive values for FDG-PET/CT by indication across 36 months

| **Setting** | **Indication** | **PPV, %** | **12 months** | | | | **24 months** | | | **36 months** | | |
| --- | --- | --- | --- | --- | --- | --- | --- | --- | --- | --- | --- | --- |
|  |  |  | **Result** | **Recurrence** | **No recurrence** | **NPV, %** | **Recurrence** | **No recurrence** | **NPV, %** | **Recurrence** | **No recurrence** | **NPV, %** |
| **STAGE 1 TESTICULAR SEMINOMA** | | | | | | | | | | | | |
| *Baseline* | *All* | NA | Positive | 0 | 0 | 96 | 0 | 0 | 95.7 | 0 | 0 | 95.5 |
|  |  |  | Negative | 4 | 96 |  | 4 | 89 |  | 4 | 84 |  |
| *Active surveillance* | *All* | 100 | Positive | 18 | 0 | 91.8 | 18 | 0 | 91.1 | 18 | 0 | 90.5 |
|  |  |  | Negative | 4 | 45 |  | 4 | 41 |  | 4 | 38 |  |
|  | *Routine* | 100 | Positive | 1 | 0 | 94.1 | 1 | 0 | 93.6 | 1 | 0 | 93.3 |
|  |  |  | Negative | 2 | 32 |  | 2 | 29 |  | 2 | 28 |  |
|  | *Clinical suspicion^#^* | 100 | Positive | 16 | 0 | 86.7 | 16 | 0 | 85.7 | 16 | 0 | 83.3 |
|  |  |  | Negative | 2 | 13 |  | 2 | 12 |  | 2 | 10 |  |
| **ADVANCED TESTICULAR SEMINOMA** | | | | | | | | | | | | |
| *Baseline* | *All* | 100 | Positive | 24 | 0 | 0 | 24 | 0 | 0 | 24 | 0 | 0 |
|  |  |  | Negative | 1 | 0 |  | 1 | 0 |  | 1 | 0 |  |
| *Follow-up* | *All* | 77.1 | Positive | 27 | 8 | 91.4 | 27 | 8 | 90.9 | 27 | 8 | 90.2 |
|  |  |  | Negative | 6 | 64 |  | 6 | 60 |  | 6 | 55 |  |
|  | *Routine* | 42.9 | Positive | 3 | 4 | 100 | 3 | 4 | 100 | 3 | 4 | 100 |
|  |  |  | Negative | 0 | 4 |  | 0 | 4 |  | 0 | 3 |  |
|  | *Clinical suspicion^#^* | 100 | Positive | 17 | 0 | 94.4 | 17 | 0 | 93.8 | 17 | 0 | 93.3 |
|  |  |  | Negative | 1 | 17 |  | 1 | 15 |  | 1 | 14 |  |
|  | *Immediate post-treatment** | 60 | Positive | 6 | 4 | 89.6 | 6 | 4 | 89.1 | 6 | 4 | 88.4 |
|  |  |  | Negative | 5 | 43 |  | 5 | 41 |  | 5 | 38 |  |

Legend: PPV= positive predictive value, NPV= negative predictive value

^#^defined by radiological change, elevation of serum tumour markers or symptoms

*Inclusive of patients both with and without residual mass post-chemotherapy
